# Anomaly detection scheme for lung CT images using vector quantized variational auto-encoder with support vector data description

**DOI:** 10.1007/s12194-024-00851-5

**Published:** 2024-10-26

**Authors:** Zhihui Gao, Ryohei Nakayama, Akiyoshi Hizukuri, Shoji Kido

**Affiliations:** 1 Graduate School of Science and Engineering, Ritsumeikan University, 1-1-1 Noji-Higashi, Kusatsu, Shiga 525-8577 Japan; 2 Institute for Radiation Sciences, Osaka University, 1-1 Yamadaoka, Suita, Osaka 565-0871 Japan

**Keywords:** Anomaly detection, Lung CT images, VQ-VAE, SVDD

## Abstract

This study aims to develop an anomaly-detection scheme for lesions in CT images. Our database consists of lung CT images obtained from 1500 examinees. It includes 1200 normal and 300 abnormal cases. In this study, SVDD (Support Vector Data Description) mapping the normal latent variables into a hypersphere as small as possible on the latent space is introduced to VQ-VAE (Vector Quantized-Variational Auto-Encoder). VQ-VAE with SVDD is constructed from two encoders, two decoders, and an embedding space. The first encoder compresses the input image into the latent-variable map, whereas the second encoder maps the normal latent variables into a hypersphere as small as possible. The first decoder then up-samples the mapped latent variables into a latent-variable map with the original size. The second decoder finally reconstructs the input image from the latent-variable map replaced by the embedding representations. The data of each examinee is classified as abnormal or normal based on the anomaly score defined as the combination of the difference between the input image and the reconstructed image and the distance between the latent variables and the center of the hypersphere. The area under the ROC curve for VQ-VAE with SVDD was 0.76, showing an improvement when compared with the conventional VAE (0.63, *p* < .001). VQ-VAE with SVDD developed in this study can yield higher anomaly-detection accuracy than the conventional VAE. The proposed method is expected to be useful for identifying examinees with lesions and reducing interpretation time in CT screening.

## Introduction

In recent years, CT (Computed Tomography) screening has become widespread to detect lung cancers at an early stage [1, 2]. A huge number of slice images obtained in CT screening causes a heavy burden on the radiologists who interpret those images. Therefore, it is desirable to develop a computerized detection scheme for lung CT images to aid diagnosis by radiologists [3–6]. There have been many studies for lesion detection in CT images using CNN (Convolutional Neural Network) based on supervised learning [7–9]. However, those detection schemes generally learn the image features of lesions of the target type and can only detect lesions of the learned target type. This detection approach would be fatal for CT screening that targets all types of lesions.

Anomaly detection is an approach that can target all kinds of abnormalities. Anomaly detection learns the image features of the normal image and then determines abnormalities based on features that differ from normal features. Therefore, anomaly detection will be able to target all types of lesions in CT-screening images. Generative models GAN (Generative Adversarial Networks) [10] and VAE (Variational Autoencoder) [11] are often used for anomaly detection. Generative models learn to dimensionally compress an input image into latent variables and reconstruct the input image from the compressed latent variables. Generative models trained using only normal images can reconstruct normal structures from latent variables but cannot accurately reconstruct abnormal structures. Abnormalities can be detected using this property [12]. However, GAN-based models with numerous parameters to optimize are challenging to train effectively [13]. Conversely, VAE with fewer parameters than GAN is relatively easier to train. VAE maps the latent variables extracted from the encoder to a normal distribution. When VAE is trained using only normal images, the probability distribution of the latent variables of untrained abnormal images would be different from that of normal images [14, 15]. Therefore, the detection accuracy of lesions with VAE is expected to be higher than that with GAN. However, the latent space of continuous representation in VAE has a problem of posterior collapse [16]. The decoder in VAE reconstructs an input image based on random sampling from the probability distribution of each latent variable obtained from the input image. If the probability distribution of all latent variables is the same as a standard normal distribution, then most of the sampling values are close to 0; this causes VAE to output a blurred reconstructed image without reflecting the features of the input image instead of accurately reconstructing normal structures.

VQ-VAE (Vector Quantized-Variational Auto-Encoder) is the model that solves this problem of VAE [17]. VQ-VAE can generate accurate reconstructed images by discretizing the continuous latent space of VAE. However, VQ-VAE is only a generative model for high-definition images and does not have a module to distance abnormal latent variables from normal latent variables. SVDD (Support Vector Data Description) is a one-class classification technique that trains to separate normal and abnormal data in a kernel space. The principle of SVDD is to map normal data into a kernel space and find a minimum hypersphere that encapsulates those data. The abnormal data are expected to be outside the hypersphere [18, 19]. In this study, a novel network (VQ-VAE with SVDD) for anomaly detection is constructed by introducing SVDD to VQ-VAE instead of a normal-distribution assumption. The VQ-VAE with SVDD is then applied to anomaly detection in lung CT-screening images.

## Materials and methods

### Materials

Our database consists of lung CT-screening images obtained from 1500 examinees at Medical Imaging Clinic (Toyonaka, Osaka, Japan). It includes 1200 normal cases and 300 abnormal cases (147 lung cancers, 60 emphysemas, 49 pneumonias, and 44 pneumothoraxes). The image sizes are 512 × 512 pixels, whereas the number of slices in each case is 72–124. The pixel size and the slice thickness are 0.55–0.86 mm and 3.75 mm, respectively. Those images are resized to 256 × 256 pixels using a bicubic interpolation. The density resolutions are also re-scaled to [0, 1] by min-max-scaling.

The proposed network is trained and tested using a sixfold cross validation test method. In the sixfold cross validation, 1200 normal cases are randomly divided into six subsets of 200 cases each. Four subsets, one subset, and the remaining one subset are used as the training dataset, validation dataset, and test dataset, respectively. The proposed network is trained and evaluated six times until each of the six subsets is used once as the test dataset. Note that all the 300 abnormal cases are used as the test dataset six times.

The proposed method is developed and evaluated using PyTorch on a workstation (central processing unit: Intel Core i9-10900X processor, random-access memory: 128 GB, and graphics processing unit: NVIDIA RTX 3090).

### VQ-VAE with SVDD

**Fig. 1:**
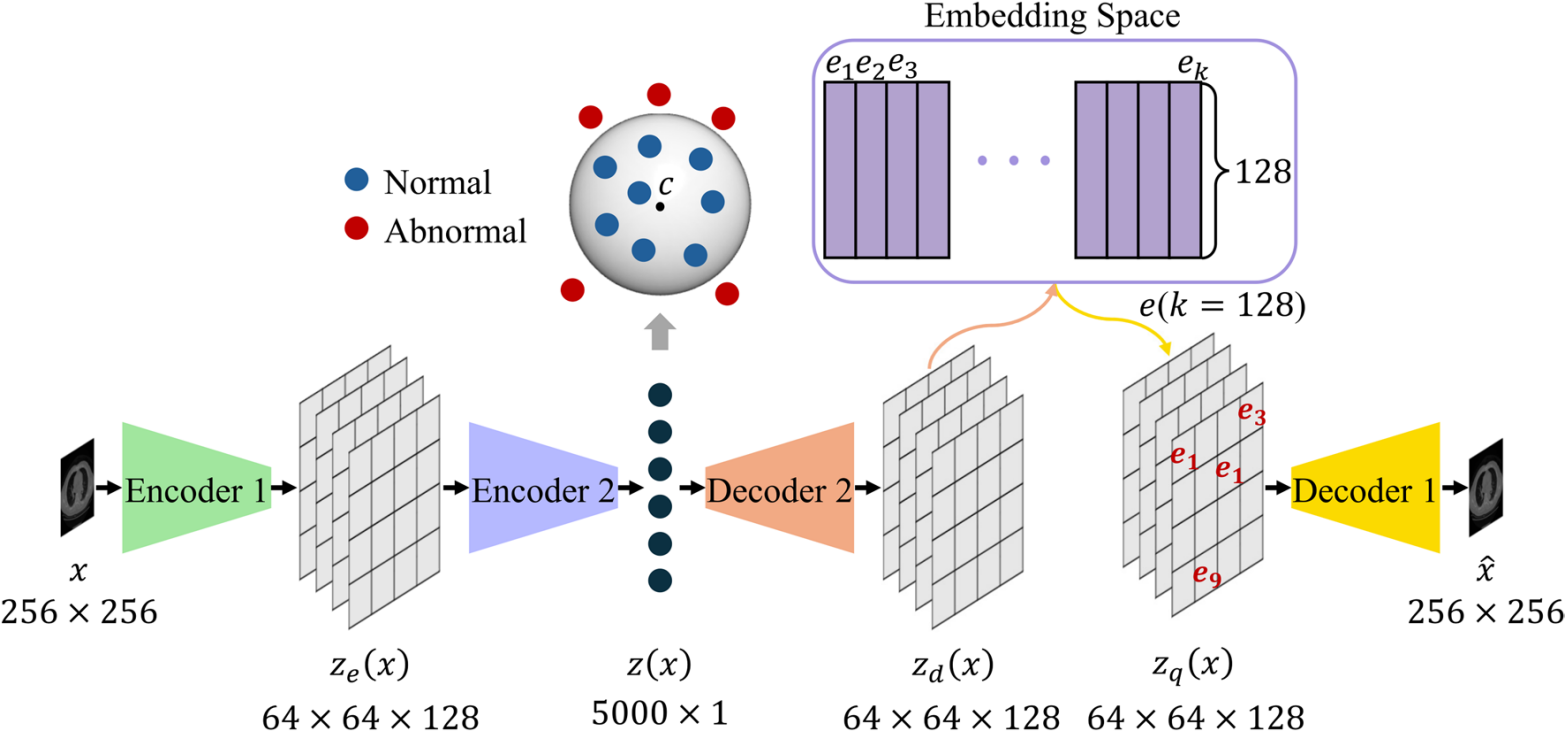
Architecture of VQ-VAE with SVDD

In this study, VQ-VAE with SVDD for anomaly detection is constructed by introducing SVDD to VQ-VAE. Figure 1 shows the architecture of VQ-VAE with SVDD. Original VQ-VAE consists of Encoder 1, Decoder 1, and Embedding Space. VQ-VAE can generate an accurate reconstructed image by replacing the continuous latent space of VAE with a discrete embedding space. However, VQ-VAE is an image-generation model, not an anomaly-detection model. SVDD for mapping the normal latent variables into a hypersphere as small as possible on the latent space is introduced to VQ-VAE to apply VQ-VAE to anomaly detection. In VQ-VAE with SVDD, Encoder 2 and Decoder 2 are added between Encoder 1 and Decoder 1 in VQ-VAE. SVDD employs generally kernel functions to project normal latent variables onto latent space. In this study, learnable Encoder 2 instead of a kernel function is used to make the projective transformation more suitable for the training data [20, 21].

**Fig. 2:**
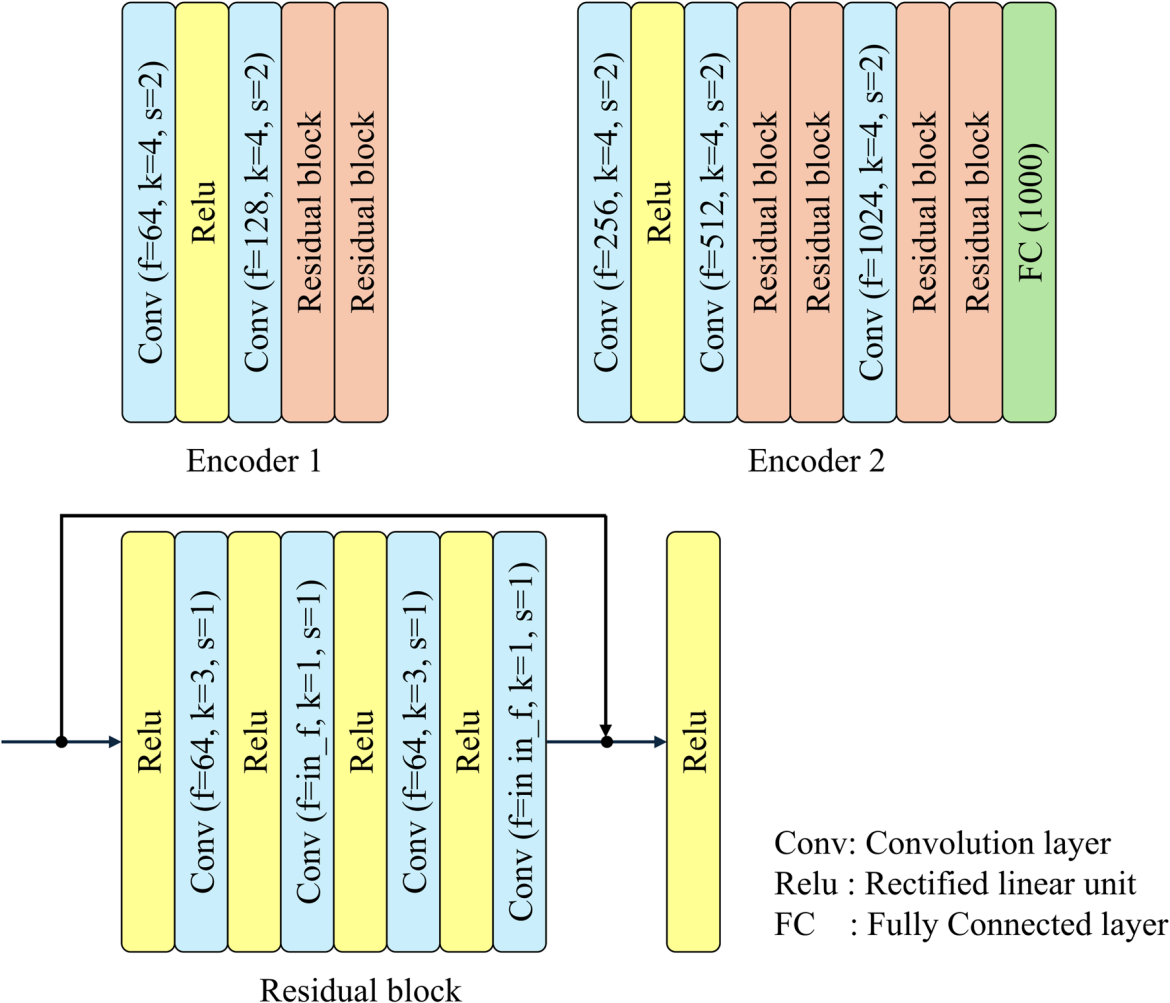
Architectures of Encoders 1 and 2

**Fig. 3:**
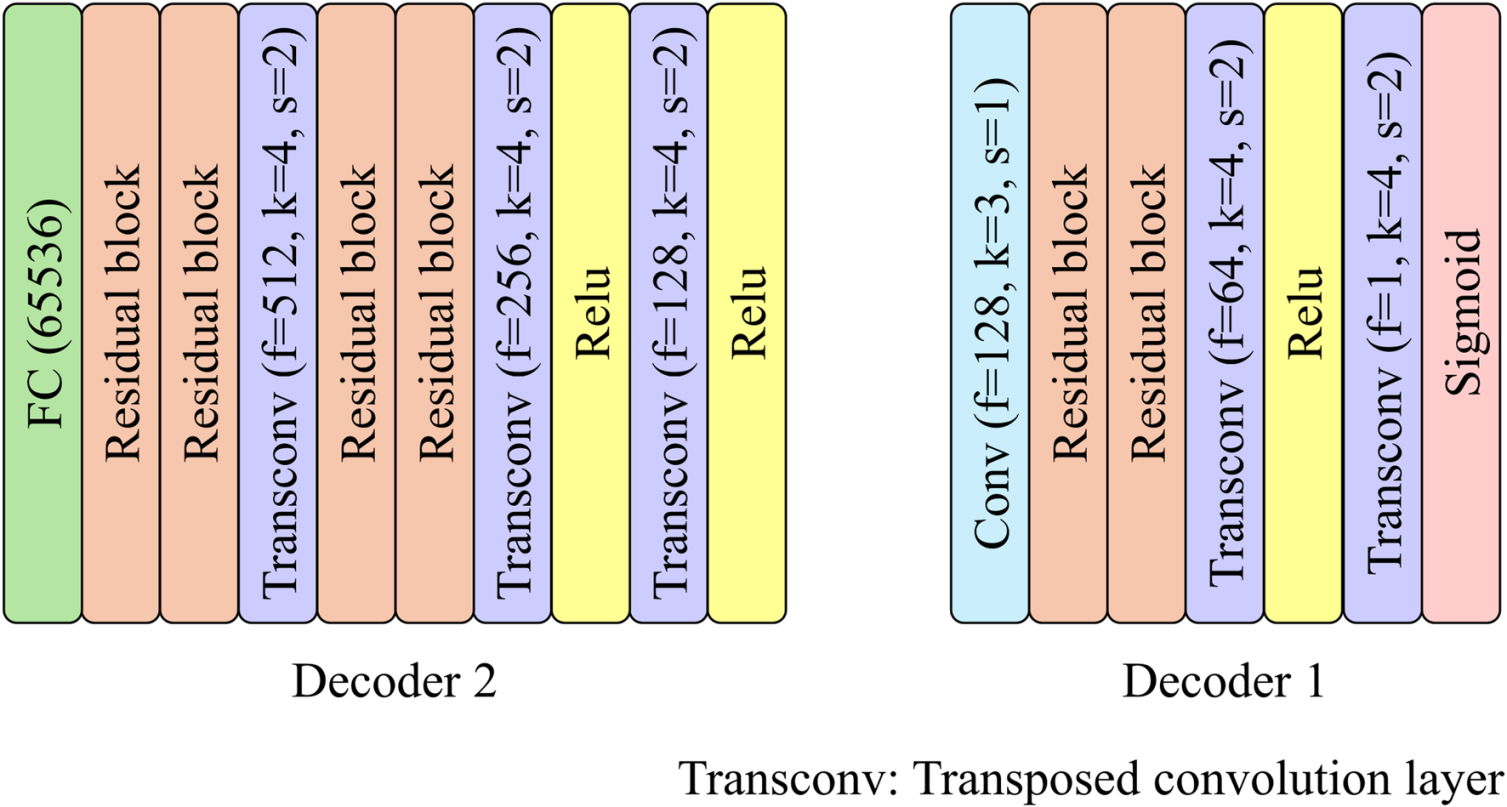
Architectures of Decoders 1 and 2

Figures 2 and 3 illustrate the architectures of Encoders 1, 2, Decoders 1, and 2. Characters f, k, and s in parentheses represent the number of filters, kernel size, and stride size, respectively. Character in_f in the residual block is the number of filters inputted to the block. The number of embedding representations in the Embedding Space is set to 128, whereas the dimension of each representation is set to 128. Encoder 1 compresses an input image $x$ to the latent-variable map ${z}_{e} (x)\in {R}^{H\times W\times D}$. $H$ and $W$ are the height and width of the latent-variable map, whereas $D$ is the number of channels. Encoder 2 also compresses the dimensionally of ${z}_{e}\left(x\right)$ to the latent variables $z(x)$ while mapping those to a hypersphere as small as possible on the latent space. Decoder 2 then up-samples $z(x)$ to the latent-variable map ${z}_{d}\left(x\right)$ with the same size as ${z}_{e}\left(x\right)$. A newly latent-variable map ${z}_{q}\left(x\right)$ is obtained by replacing the latent variables in the latent-variable map ${z}_{d}\left(x\right)$ with the embedding representations ${e}_{k}$ in the Embedding Space. The distances are first determined between the channel direction vectors at each pixel in ${z}_{d}\left(x\right)$ and all ${e}_{k}$ in this replacement defined by Eq. (1). The closest embedding representation is then selected and placed at each corresponding pixel in ${z}_{q}\left(x\right)$.

1 $$\begin{array}{c}{z}_{q}\left(x\right)={e}_{k}, \quad where\quad k={\text{argmin}}_{j}{\Vert {z}_{d}\left(x\right)-{e}_{j}\Vert }_{2}\end{array}$$

Decoder 2 reconstructs the image $\widehat{x}$ from ${z}_{q}\left(x\right)$.

### Training of VQ-VAE with SVDD

VQ-VAE with SVDD is trained by the following three steps:

**Step 1:** The embedding space must be trained to reflect the features of the latent variables created from normal CT images. Only the VQ-VAE part consisting of Encoder 1, Decoder 1, and Embedding Space is first trained with the loss function Eq. (2). Note that ${z}_{e}\left(x\right)$ in Fig. 1 does not pass through Encoder 2 and Decoder 2 and becomes ${z}_{d}\left(x\right)$.

2 $$\begin{array}{c}{\mathcal{L}}_{VQ-VAE}={\Vert x-\widehat{x}\Vert }_{2}^{2}+\left(1-MS\_SSIM(x, \widehat{x})\right)+{\Vert sg\left[{z}_{e}\left(x\right)\right]-e\Vert }_{2}^{2}+\lambda {\Vert {z}_{e}\left(x\right)-sg\left[e\right]\Vert }_{2}^{2}\end{array}$$

The first term represents the L2 norm between the input image $x$ and the reconstructed image $\widehat{x}$. MS-SSIM (Multi Scale-Structural Similarity) [22, 23] in the second term measures the similarity of brightness, contrast, and structure between two images. MS-SSIM is defined by Eq. (3).

3 $$\begin{array}{c}MS\_SSIM(x, \widehat{x})={\left[L\left(x, \widehat{x}\right)\right]}^{\alpha }\cdot\prod_{j=0}^{M}{(\left[{C}_{j}\left(x,\widehat{x}\right)\right]}^{\beta }\cdot {\left[{S}_{j}\left(x,\widehat{x}\right)\right]}^{\gamma })\end{array}$$

The terms for brightness $L\left(x,\widehat{x}\right)$, contrast $C(x,\widehat{x})$, and structure $S(x,\widehat{x})$ are defined by the following equations:

4 $$\begin{array}{c}L\left(x,\widehat{x}\right)=\frac{\left(2{\mu }_{x} {\mu }_{\widehat{x}}+{\delta }_{1}\right)}{\left({\mu }_{x}^{2}+{\mu }_{\widehat{x}}^{2}+{\delta }_{1}\right)}, C\left(x,\widehat{x}\right)=\frac{\left(2{\sigma }_{x} {\sigma }_{\widehat{x}}+{\delta }_{2}\right)}{\left({\sigma }_{x}^{2}+{\sigma }_{\widehat{x}}^{2}+{\delta }_{2}\right)}, S\left(x,\widehat{x}\right)=\frac{\left(2{\sigma }_{x\widehat{x}}+{\delta }_{3}\right)}{\left({\sigma }_{x}+{\sigma }_{\widehat{x}}+{\delta }_{3}\right)}\end{array}$$

$j$ indicates the number of times $x$ and $\widehat{x}$ are downsampled to the size of 1/2. Contrast ${C}_{j}(x,\widehat{x})$ and structure ${S}_{j}(x,\widehat{x})$ are determined at different resolutions $j$. ${\mu }_{x}$, ${\mu }_{\widehat{x}}$, ${\sigma }_{x}$, ${\sigma }_{\widehat{x}}$, ${\sigma }_{x\widehat{x}}$ are mean, standard deviation, mutual covariance of $x$ and $\widehat{x}$, whereas ${\delta }_{1}$, ${\delta }_{2}$, ${\delta }_{3}$ are the normalization constants. In this study, ${\delta }_{1}$, ${\delta }_{2}$, and ${\delta }_{3}$ are given by 0.0001, 0.0009, and 0.00045, respectively. $\alpha$, $\beta$, and $\gamma$ are set to 1, whereas $M$ is set to 5. In the third and fourth terms of Eq. (2), sg (stop gradient) is the operator that does not compute the gradient in error backpropagation. When each latent variable in ${z}_{e}\left(x\right)$ is replaced with the embedding representation ${e}_{k}$, the distances between the latent variables and the embedding representations are determined in the embedding space. This process is not differentiable. The third term updates the embedding representations ${e}_{k}$ to close to ${z}_{e}\left(x\right)$. Note that the gradient of this term is not propagated to Encoder 1. The fourth term contributes to updating Encoder 1 to close ${z}_{e}\left(x\right)$ to ${e}_{k}$. Note that the gradient of this term is unchangingly propagated to Encoder 1. The coefficient $\lambda$ is set to 0.25 empirically.

**Step 2:** To determine an initial hypersphere containing only normal latent variables extracted from normal CT images, the proposed network without SVDD is trained using the loss function of Eq. (5). The initial values of Encoder 1, Embedding Space, and Decoder 1 are given by the weights learned in Step 1. Note that Encoder 2 does not train to map the normal latent variables into a hypersphere as small as possible.

5 $${\mathcal{L}}_{Center} = {\Vert x-\widehat{x}\Vert }_{2}^{2} + \left( {1 - MS\_SSIM\left( {x, \hat{x}} \right)} \right) + {\Vert sg\left[ {z_{d} \left( x \right)} \right] - e \Vert }_{2}^{2} \begin{array}{*{20}c} { + \lambda {\Vert z_{d} \left( x \right) - sg\left[ e \right] \Vert }_{2}^{2} + {\Vert z_{e} \left( x \right) - z_{d} \left( x \right) \Vert }_{2}^{2} } \\ \end{array}$$

The first to fourth terms correspond to those in Eq. (2). The fifth term is the L2 norm between the latent-variable maps ${z}_{e}\left(x\right)$ input to Encoder 2 and ${z}_{d}(x)$ output from Decoder 2. The center of the initial hypersphere for SVDD is then determined by the average latent variables $z(x)$ output from Encoder 2 in the trained network without SVDD.

**Step 3:** The initial values of VQ-VAE with SVDD are given by the weights learned in Step 2. The entire proposed network with SVDD is trained using the loss function of Eq. (6). The center of the hypersphere is updated every epoch by the average latent variables $z\left(x\right)$ output from Encoder 2.

6 $${\mathcal{L}}_{VQ - SVDD} = {\Vert x-\widehat{x}\Vert }_{2}^{2} + \left( {1 - MS\_SSIM\left( {x, \hat{x}} \right)} \right) + {\Vert sg\left[ {z_{d} \left( x \right)} \right] - e \Vert }_{2}^{2} \begin{array}{*{20}c} { + \lambda {\Vert z_{d} \left( x \right) - sg\left[ e \right] \Vert }_{2}^{2} + {\Vert z_{e} \left( x \right) - z_{d} \left( x \right) \Vert }_{2}^{2} } + {\Vert z\left( x \right) - c \Vert }_{2}^{2} \\ \end{array}$$

The first to fifth terms correspond to those in Eq. (5). The sixth term contributes to updating Encoder 2 to close the latent variables $z(x)$ to the center $c$ of the hypersphere.

The same training dataset consisting of only normal CT images is used for training of Step 1, Step 2, and Step 3. The hyperparameters for training are set with a batch size of 64, a learning rate of $1e-4$, and a maximum epoch number of 200. In all steps, Adam is employed to optimize network weights.

### Anomaly score and evaluation indices

Anomaly score $S(x)$ at each slice image is defined as the combination of the difference between the input image $x$ and the reconstructed image $\widehat{x}$ and the distance between the latent variables $z\left(x\right)$ and the center $c$ of the hypersphere.

7 $$\begin{array}{c}S\left(x\right)={\Vert x-\widehat{x}\Vert }_{2}^{2}+{\Vert z\left(x\right)-c\Vert }_{2}^{2}\end{array}$$

The maximum anomaly score in all slice images for each examinee is determined as the representative score. If the representative anomaly score is higher than a threshold value, the examinee is classified as abnormal. Here, the threshold value is set to maximize the Youden index based on Receiving Operating Characteristic (ROC) analysis for all the representative scores. The classification accuracy, sensitivity, the specificity are also evaluated based on the representative anomaly scores.

The fidelity of the reconstructed images is compared between normal and abnormal images, as well as between a conventional VAE and the proposed model. In this study, the root mean squared error (RMSE), peak signal to noise ratio (PSNR), and structural similarity index measure (SSIM) are employed as evaluation metrics [24, 25].

These evaluation metrics are determined for each subset of the sixfold cross validation test. Then, the significance is assessed using a paired t-test.

## Results

**Fig. 4:**
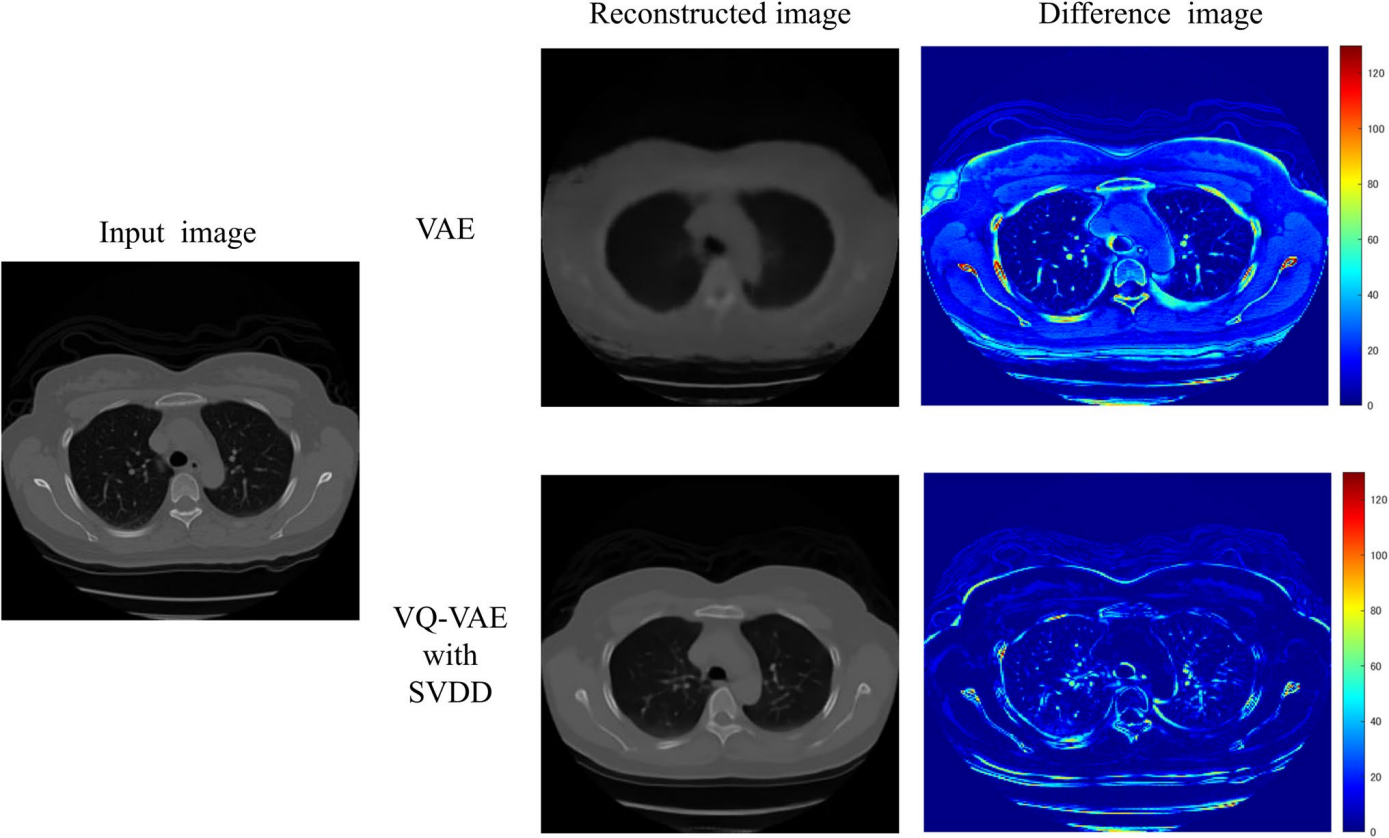
Reconstructed images for the normal image with VAE and the proposed method

**Fig. 5:**
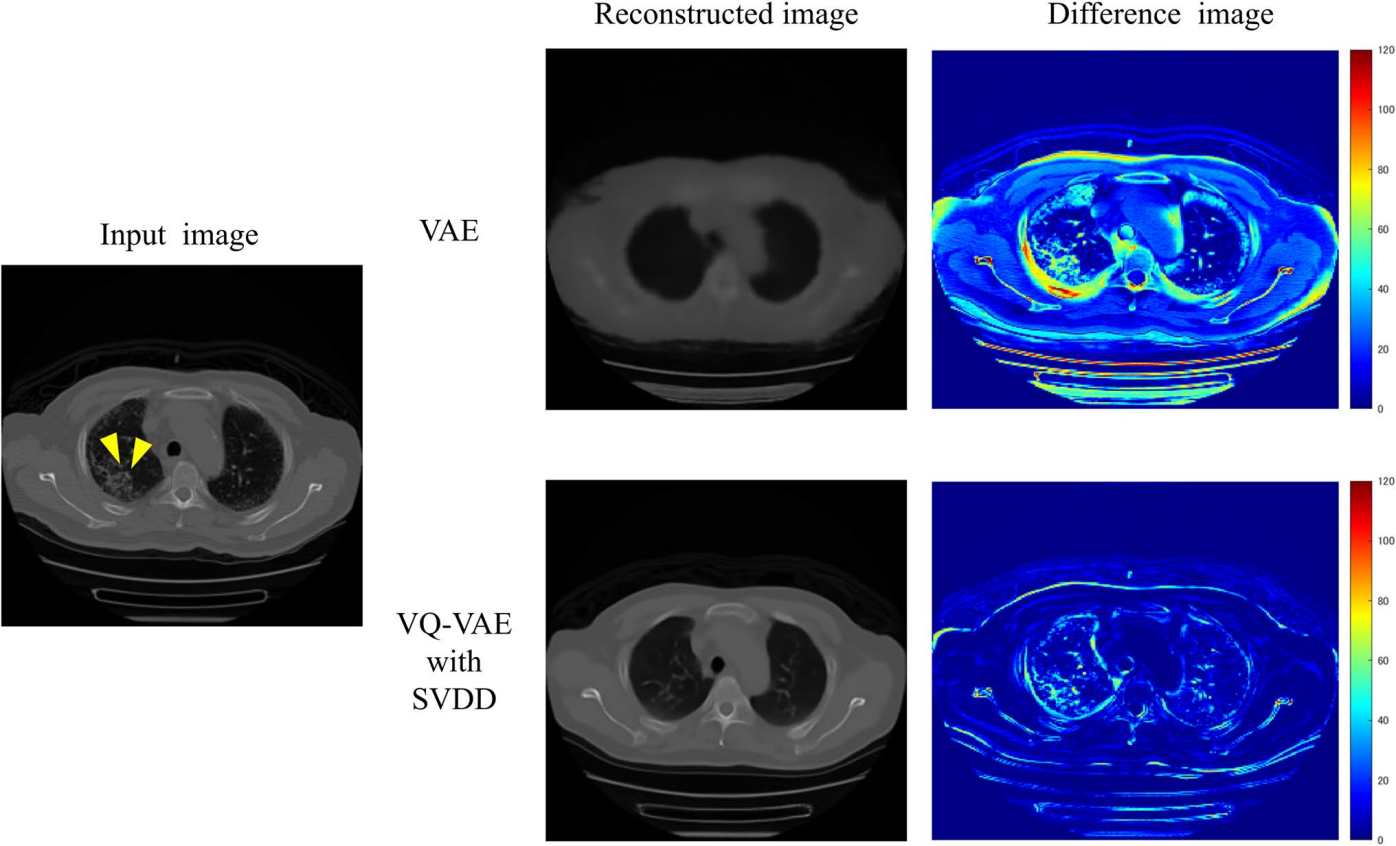
Reconstructed images for the abnormal image with VAE and the proposed method

Figure 4 compares the reconstructed images for the normal CT image by a traditional VAE and VQ-VAE with SVDD. The reconstructed image with VAE was blurred, and the normal structures could not be restored. Conversely, the blurring of the reconstructed image with the proposed method was suppressed, and the normal structures could be restored more accurately. Figure 5 compares the reconstructed images for the abnormal CT image by two different models. The lesion indicated by the arrow was not restored with either method. Table 1 shows the fidelities of the reconstructed images for normal and abnormal images by VAE and VQ-VAE with SVDD. Note that the fidelity for normal images was evaluated from all slices of normal cases, whereas that for abnormal images was evaluated only from slices containing abnormalities. In both normal and abnormal images, the proposed model showed substantial improvements in the mean SSIM, mean PSNR, and mean RMSE compared to the VAE. With the proposed model, the mean SSIM (0.78), mean PSNR (27.30), and mean RMSE (0.044) for abnormal images were significantly deteriorated than those for normal images (0.83, *p* < 0.001; 28.74, *p* < 0.001; 0.037, *p* < 0.001).

**Table 1:** Fidelities of the reconstructed images for normal and abnormal images by VAE and VQ-VAE with SVDD

	Type	SSIM	PSNR	RMSE
VAE	normal	0.75±0.008	26.71±0.45	0.047±0.0017
	abnormal	0.72±0.014	25.31±0.49	0.048±0.0027
VQ-VAE with SVDD	normal	0.83±0.018	28.74±0.71	0.037±0.0037
	abnormal	0.78±0.015	27.30±0.59	0.044±0.0029

Table 2 illustrates the anomaly-detection performance for the test dataset by VAE and VQ-VAE with SVDD. The mean-classification accuracy, mean sensitivity, mean specificity, and mean AUC for VQ-VAE with SVDD were 0.71, 0.68, 0.73, and 0.76, respectively, showing an improvement substantially when compared with the conventional VAE (0.62, *p* < 0.001; 0.63, *p* < 0.001; 0.61, *p* < 0.001; 0.63, *p* < 0.001). Table 3 shows the mean sensitivities for each disease by VAE and VQ-VAE with SVDD. The sensitivity for emphysema by VQ-VAE with SVDD was 0.66, slightly lower than that by VAE (0.68). However, the sensitivity for lung cancer (0.59), pneumonia (0.58), and pneumothorax (0.73) were improved by VQ-VAE with SVDD (0.70, 0.63, and 0.76).

**Table 2:** Anomaly detection performance by VAE and VQ-VAE with SVDD

	Accuracy	Sensitivity	Specificity	AUC
VAE	0.62±0.015	0.63±0.038	0.61±0.045	0.63±0.013
VQ-VAE with SVDD	0.71±0.017	0.68±0.040	0.73±0.046	0.76±0.014

**Table 3:** Sensitivities for each disease by VAE and VQ-VAE with SVDD

	Lung cancer	Emphysema	Pneumonia	Pneumothorax
VAE	0.59±0.035	0.68±0.050	0.58±0.046	0.73±0.021
VQ-VAE with SVDD	0.70±0.032	0.66±0.048	0.63±0.053	0.76±0.023

## Discussion

In this study, VQ-VAE with SVDD for anomaly detection was constructed by introducing SVDD to VQ-VAE to improve the accuracy of the reconstructed images and the anomaly-detection performance. Using the proposed model, the normal structures can be restored more accurately in the reconstructed images while suppressing the restoration of abnormal structures. The proposed method can also achieve higher anomaly-detection performance than the traditional VAE. VQ-VAE with SVDD, which solves the problems of VAE, might contribute to explainable AI (Artificial Intelligence). To the best of our knowledge, anomaly-detection approaches that combine VQ-VAE and SVDD have not been proposed before.

VAE could hardly restore the shape of the body and organs in the reconstructed images, as shown in Figs. 4, 5, and Table 1. In VAE, the probability distribution of latent variables in latent space is assumed to be a standard normal distribution. The output values from the encoder in VAE are set as the mean value and the standard deviation for the probability distribution of each latent variable. The newly latent variables sampled from the probability distribution of each latent variable are input to the decoder for reconstructing the image. As training progresses, the probability distributions of all latent variables are sometimes close to the standard normal distribution; this causes the sampled latent variables to have similar values. The latent variables used for image reconstruction should vary greatly since the location and shape of organs and the distribution of blood vessels in CT images vary. Therefore, images reconstructed from similar latent variables will not have the detailed shapes restored, resulting in blurred images. Conversely, discrete embedding representations are used in image reconstruction of VQ-VAE instead of latent variables sampled from probability distributions. Embedded representations do not become similar through training. Those are also updated to improve the accuracy of image reconstruction. Therefore, VQ-VAE can be expected to provide more accurate image reconstruction than VAE.

Original VQ-VAE consists of Encoder 1, Decoder 1, and Embedding Space. If the size of the latent-variable map from Encoder 1 becomes too small for the input image, the reconstruction accuracy will reduce substantially since detailed information on structures will be lost. Therefore, it would be preferable to keep the latent-variable map from Encoder 1 to one-fourth of the input image size, following the paper [17]. The size of the latent-variable map from Encoder 1 used in this study is 64 × 64 × 128. Even if SVDD is applied to each latent variable in the latent-variable map, a lot of redundant information for reconstructing normal structures will remain because the number of elements in the latent-variable map is significantly larger than the input image with 256 × 256 pixels. When an abnormal image is input, the abnormal structures will be unexpectedly restored from the redundant information. Therefore, Encoder 2 and Decoder 2 are added to the original VQ-VAE in this study to introduce SVDD to VQ-VAE effectively. Encoder 2 compresses the latent variables into a hypersphere as small as possible on the latent space while preserving the information necessary to restore normal structures. Decoder 2 reconstructs the latent-variable map as close as possible to the input of Encoder 2 from the compressed latent variables in training with the fifth term in Eq. (5). The number of elements in the compressed latent variables is sufficiently smaller than the input image, and there will be almost no redundant information. Therefore, VQ-VAE with SVDD can be expected to suppress the restoration of abnormal structures.

If the entire proposed network is trained at once, information about fine structures like blood vessels in the latent variables would be lost due to dimensionality reduction by Encoders 1 and 2. Step-by-step learning is adopted in this study. Only the VQ-VAE part consisting of Encoder 1, Decoder 1, and Embedding Space is first trained to restore normal structures accurately. Through this training, the embedded representations are updated to be suitable for restoring normal structures. For subsequent training, the L2 norm of ${z}_{e}\left(x\right)$ and ${z}_{d}\left(x\right)$ is added to the loss function so that the trained embedding representations are used effectively. Mean squared error and L2 norm between images are generally used as the loss function in training image reconstruction. Those indices focus on differences in pixel values between images and less on structures. The location and shape of organs and the distribution of blood vessels in CT images vary. Therefore, MS-SSIM evaluating structures at different resolutions is added to the loss function. Step-by-step learning and MS-SSIM will improve the reconstruction accuracy of the proposed model.

**Fig. 6:**
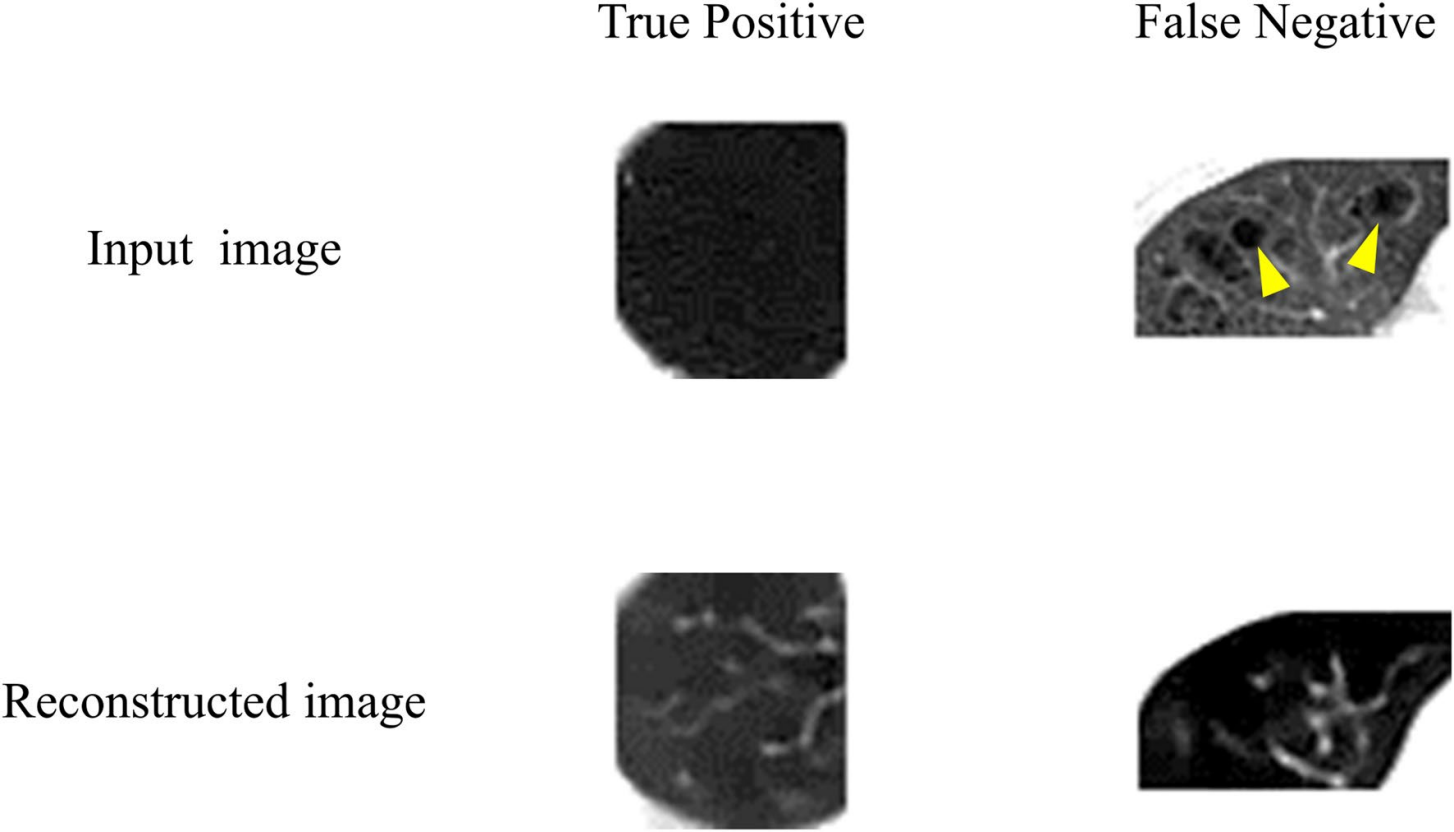
Example of reconstructed images for emphysema with the proposed method

The sensitivity for emphysema with the proposed method was lower than that with VAE, as shown in Table 3. In emphysema, collapsed alveoli appear black without clear structures on CT images. The proposed model trained using only normal images reconstructs likely normal structures instead of the lesions. When emphysema spread, as in the true positive case in Fig. 6, structures like blood vessels were reconstructed with the proposed model. With the anomaly score based on the difference between input and output images, this image was classified as abnormal. Conversely, no obvious structures were reconstructed in the lesion areas when emphysema is localized, as illustrated in the false negative case in Fig. 6. We consider this to be due to the localization of areas with no clear structure, even in normal lung fields. In further study, it will be necessary to optimize the weights for the combination of the difference between the input image and the reconstructed image and the distance between the latent variables and the center of the hypersphere in the anomaly score.

To verify the validity for the number of training data, Table 4 shows AUCs by VAE and VQ-VAE with SVDD trained on different numbers of training data. The AUCs for VAE and VQ-VAE with SVDD trained on 200 training cases were 0.52 and 0.71, respectively, which were significantly improved to 0.64 and 0.75 by using 400 training cases. However, those changed little even when the training data was increased to 600 or 800 cases. Since the purpose of VAE is basically to learn the probability distribution of latent variables, about 400 cases would have been sufficient for that training. We believe that the low AUC for VAE is not due to the number of training data, but to the architecture of VAE, which cannot reconstruct detailed shapes. VQ-VAE with SVDD, which uses discrete embedding representations instead of latent variables in image reconstruction, was able to learn relatively well with even less training data of 200 cases. Although VQ-VAE with SVDD maps the normal latent variables into a hypersphere as small as possible on the latent space, the hypersphere will not be updated if only latent variables similar to those in the hypersphere are obtained from added training images. Therefore, even if the training data was increased to 600 or 800 cases, the AUC for VQ-VAE with SVDD did not improve significantly. However, the AUC improved slightly as the training data increases, so increasing the training data further might yield a higher AUC.

**Table 4:** AUCs by VAE and VQ-VAE with SVDD trained on different numbers of training data

	200 cases	400 cases	600 cases	800 cases
VAE	0.52	0.64	0.65	0.64
VQ-VAE with SVDD	0.71	0.75	0.76	0.77

This study has a few limitations. One limitation is that CT images resized to 256 × 256 pixels were used for the input of the proposed model. It was limited to analyzing CT images with 256 × 256 pixels due to the processing performance of the used workstation. However, although the anomaly-detection performance may be improved by the use of CT images with the original size of 512 × 512 pixels, we believe that the usefulness of the proposed method would not change. Another limitation is that each examinee was classified as abnormal or normal based on the representative anomaly score. We believe that picking up examinees with abnormal lesions will be useful in lung CT screening. However, presenting abnormalities slice-by-slice and lesion-by-lesion would be more helpful in reducing the burden on radiologists. In the further study, we will address those issues.

## Conclusion

VQ-VAE with SVDD developed in this study can yield higher anomaly-detection accuracy than the conventional VAE. The proposed method is expected to be useful for identifying examinees with lesions and for reducing interpretation time in CT screening.

## Data Availability

The database used in the current study is available from the corresponding author on reasonable request.
